# Encapsulation of Plant Biocontrol Bacteria with Alginate as a Main Polymer Material

**DOI:** 10.3390/ijms222011165

**Published:** 2021-10-16

**Authors:** Roohallah Saberi Riseh, Yury A. Skorik, Vijay Kumar Thakur, Mojde Moradi Pour, Elahe Tamanadar, Shahnaz Shahidi Noghabi

**Affiliations:** 1 Department of Plant Protection, Faculty of Agriculture, Vali-e-Asr University of Rafsanjan, Imam Khomeini Square, Rafsanjan 7718897111, Iran; r.saberi@vru.ac.ir (R.S.R.); Moradi.mojde21@gmail.com (M.M.P.); E.Tamanadar72@gmail.com (E.T.); Shahidi@vru.ac.ir (S.S.N.); 2 Institute of Macromolecular Compounds of the Russian Academy of Sciences, Bolshoi VO 31, St. Petersburg 199004, Russia; 3 Biorefining and Advanced Materials Research Center, SRUC, Edinburgh EH9 3JG, UK; Vijay.Thakur@sruc.ac.uk; 4 Department of Mechanical Engineering, School of Engineering, Shiv Nadar University, Greater Noida 201314, Uttar Pradesh, India; 5 School of Engineering, University of Petroleum and Energy Studies (UPES), Dehradun 248007, Uttarakhand, India

**Keywords:** alginate, chitosan, encapsulation, pest management, plant disease

## Abstract

One of the most favored trends in modern agriculture is biological control. However, many reports show that survival of biocontrol bacteria is poor in host plants. Providing biocontrol agents with protection by encapsulation within external coatings has therefore become a popular idea. Various techniques, including extrusion, spray drying, and emulsion, have been introduced for encapsulation of biocontrol bacteria. One commonly used biopolymer for this type of microencapsulation is alginate, a biopolymer extracted from seaweed. Recent progress has resulted in the production of alginate-based microcapsules that meet key bacterial encapsulation requirements, including biocompatibility, biodegradability, and support of long-term survival and function. However, more studies are needed regarding the effect of encapsulation on protective bacteria and their targeted release in organic crop production systems. Most importantly, the efficacy of alginate use for the encapsulation of biocontrol bacteria in pest and disease management requires further verification. Achieving a new formulation based on biodegradable polymers can have significant effects on increasing the quantity and quality of agricultural products.

## 1. Introduction

**Figure 1:**
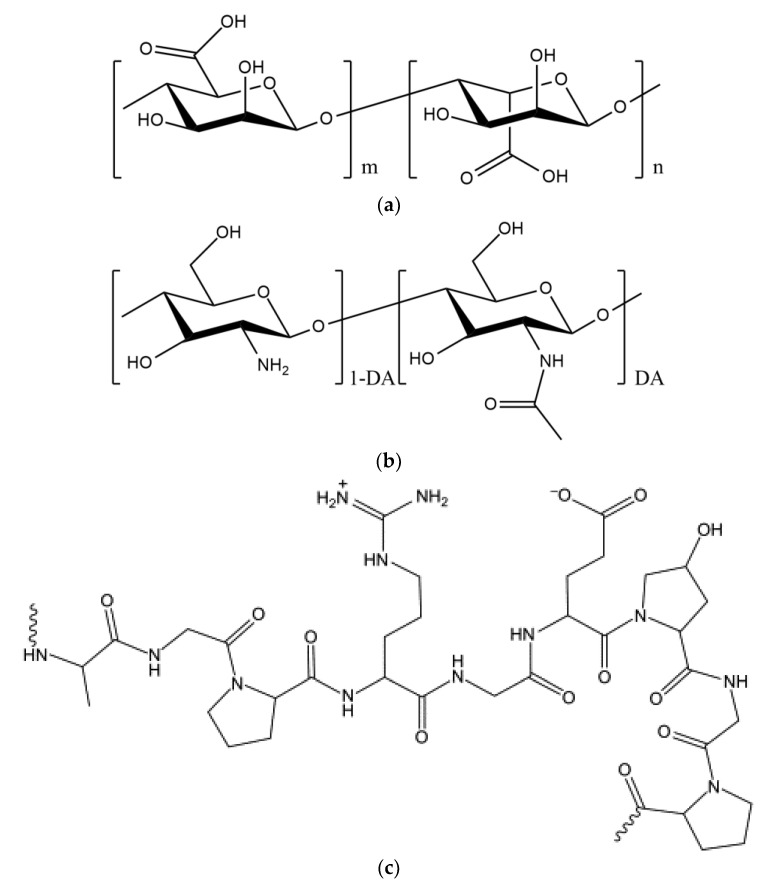
Chemical structures of: (**a**) alginic acid; (**b**) chitin (degree of acetylation, DA ≥ 0.5) and chitosan (DA < 0.5); and (**c**) gelatin.

Sodium alginate (ALG) is a polysaccharide found abundantly in the cell walls of brown algae (e.g., *Macrocystis pyrifera, Ascophyllum nodosum,* and *Sargassum sinicola*) and some bacterial species (e.g., *Pseudomonas* and *Azotobacter*) [1]. ALG has a similar role in seaweed to that of cellulose in plants and occurs as a mixture of insoluble calcium, magnesium, potassium, and sodium salts in the cell walls of seaweeds. ALG is a linear copoly–mer with homopolymeric blocks of (1→4)-linked β-d-mannuronate (M) and α-l-guluronate (G) residues linked together in different sequences (Figure 1a). The monomers may appear in homopolymeric blocks of consecutive G residues, consecutive M residues, or alternating M and G residues [2]. The ALG molecule carries a net negative charge due to the presence of carboxylate groups in both the M and G residues. ALG is soluble in water and is formed by the dissolution of a liquid with a high viscosity [3]. This substance is a type of gum that can be dissolved in both cold and hot water and that forms irreversible gels when it reacts with calcium salts or acids [4]. The properties of ALG vary depending on its origin, and they change with the M/G ratio and the molecular weight (MW) [5]. The effect of the MW of ALG is ambiguous, and optimization is required for a specific application and a specific technology. For example, increasing the MW of ALG improves the mechanical properties of the subsequent gels; however, a solution formed from high-MW ALG becomes very viscous, a condition that is mostly unfavorable in processes such as encapsulation [6]. The MW of ALG affects both its physicochemical and its biological properties. For example, Zhang et al. [7] have shown that ALG oligosaccharides with an average degree of polymerization in the range of 4.2–11.4 can effectively enhance the resistance of rice (*Oryza saliva* L.) plants to the pathogen *Magnaporthe grisea* by acting as functional elicitor of phenylalanine ammonia lyase, peroxidase, and catalase in the rice cells.

ALG has many industrial applications, including stabilization of viscosity, increasing the viscosity of gels, storage and transfer of various drugs and biomolecules, and water retention [8]. Numerous studies have been conducted on the use of ALG in pest and disease control. For example, mixing the herbicide paraquat with ALG nanoparticles increases the herbicide release properties and interaction with the soil, while reducing the negative effects of paraquat, such as the reduction of nontarget species and the influx of other species, thereby making the herbicide more effective [8]. ALG nanoparticles can also serve as carrier systems for herbicides, as ALG regulates the release of various substances, including vaccines, proteins, and different drugs [9,10].

One of the basic uses of ALG is in encapsulation technology, and one important use of encapsulation using biodegradable beads is entrapment of plant growth-promoting rhizo bacteria (PGPR) to improve conditions for the survival of these important bacterial species [11]. Plants can grow better in the presence of plant probiotic bacteria, as these bacteria carry out a number of different important functions, including nitrogen fixation, siderophore production, and mineral dissolution, and they also synthesize auxin, cytokinin, vitamins, and similar substances that are essential for plant growth [12]. However, PGPR do not readily colonize around plant roots due to their sensitivity to different soil attributes, such as humidity, temperature, pH, competition, and environmental stress [13]. Encapsulation of these microorganisms in microcapsules therefore has significant survival benefit, while also functioning to ensure the controlled release of these bacteria throughout the growing season [14]. Favorable encapsulation of viable microbial cells requires a protocol that maintains bacterial viability throughout the various handling and storage processes, as well as the use of an encapsulation material compatible with the bacteria [15].

ALG has been frequently applied as a perfect substrate for bacterial encapsulation due to its great environmental biodegradability and compatibility [16]. Among its many uses, its utilization in encapsulating bacterial agents that have important roles in managing pests and plant diseases has become one of its most important applications. One advantage of using ALG for encapsulation is that the gel formation process is conducted under ambient room conditions [17], and another is that bacterial cells can readily diffuse into the tiny ALG pores and become entrapped there. Also, the advantages of using ALG in formulations are its slow release of encapsulated microorganisms into the soil and its non-toxic nature, biodegradability, low cost, and resistance to acidic environments [18]. However, some disadvantages of ALG can create difficulties in its use. For instance, chelating agents, including phosphate and citrate, or anti-gelling cations, such as Mg^2+^ and Na^+^, are often used in biological applications and may decrease the stability of ALG gels [3]. Mixing ALG with other polymers can resolve this problem, and some researchers have used this solution (see Table 1 and Table 2).

One of the most useful combinations of polymers for various release systems is a combination of ALG and chitosan [38,39,40]. Chitosan is produced by the complete or partial deacetylation of chitin and consists of randomly distributed β-(1→4)-linked d-glucosamine and *N*-acetyl-d-glucosamine residues (Figure 1b). The degree of acetylation (DA) of chitosan ranges from 0% to 50%. Protonation of the chitosan amino groups in dilute aqueous solutions of many organic and mineral acids imparts solubility, but chitosan remains insoluble in alkaline or neutral media. The solubility of chitosan also depends on its MW and DA, as chitosans with lower MW and DA are more soluble [41]. Chitosan is a biocompatible, non-toxic, and biodegradable polymer that has been recognized as an excellent biopolymer for improving ALG capsule coatings [16,42]. The electrostatic interactions between the carboxylic groups of ALG and the protonated amino groups of chitosan cause the formation of polyelectrolyte complexes with different structures and properties [43]. The controlled release of different chemical or biological agents is possible with the use of ALG and chitosan polyelectrolyte complexes in microcapsule form [44]. Some studies have revealed that *Lactobacillus plantarum* [45], *L. bulgaricus* [46], and *Bifidobacterium longum* [47] have greater storage viability in ALG-chitosan microcapsules compared to free bacterial cells.

Another prospective companion polymer for ALG is gelatin, a protein that consists of a large number of glycine, proline, and 4-hydroxyproline residues, as well as other amino acids (Figure 1c). The amino acid composition of gelatin varies, especially in terms of its minor constituents, depending on the source of the raw material and the processing technique [48]. Its thickening and gel-like properties have led to a wide use of gelatin in the food, pharmaceutical, photography, and even cosmetics industries. The best type of gelatin is pure gelatin powder, which is made from the skins and bones of animals, such as cattle and sheep [49]. Gelatin is insoluble in cold water but readily dissolves in hot water. It absorbs up to 10 times its volume of water and forms a gel at 40 to 50 °C [49]. Due to the abundance, low cost, and biodegradability of gelatin, many researchers have tried to combine it with ALG for the preparation of microcapsules [29].

The main purpose of this review is to introduce an effective technology for the encapsulation of bacteria known to be effective in the management of pests and plant diseases.

## 2. Biological Control of Plant Diseases

**Figure 2:**
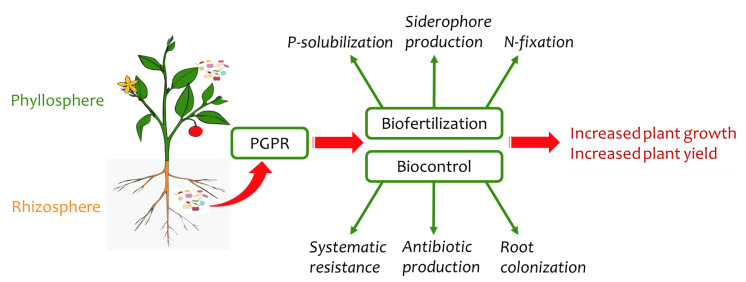
Plant probiotic bacteria and their mechanism of action (adapted from [62]).

Not only is chemical control uneconomical, it often does not show the necessary effectiveness against soil pathogens [50]. Furthermore, the continued use of chemical control methods favors the development of resistant pathogens, while also negatively affecting the quality of food products and the environment. Over the past two decades, the use of plant probiotic bacteria has emerged as a highly promising new strategy for controlling plant pathogens [51] as part of the drive for healthy, low-cost, and low-risk methods for integrated plant-disease management. The application of PGPR, which are rhizobacteria that colonize the surface of the root (Figure 2), is one of the methods that, unlike chemical control methods, leaves no toxic residues. The PGPR organisms also have beneficial interactions with the plant and increase plant growth while exerting their antagonistic activity against pathogens in the soil [52]. The PGPR genera *Arthrobacter*, *Enterobacter, Azospirillum, Azotobacter, Streptomyces, Serratia, Pseudomonas, Bacillus, Rhizobium, Klebsiella,* and *Burkholderia* are typically present in the rhizosphere [51].

Biocontrol of plant diseases is defined as a decrease in the activity of a pathogen by interaction with one or more beneficial microorganisms or antagonists [53]. *Pseudomonas fluorescens* and *Bacillus* species are used in the rhizosphere as bacterial biocontrol agents and are ideal candidates for increasing the growth of plants and controlling plant diseases under in situ and in vivo conditions [54]. For example, the functional mechanism of *B. cereus* bacteria against potato dry rot involves the production of volatiles and enzymes that degrade chitin and glucan, with a subsequent 66–89% reduction in disease [55]. *B. subtilis* is known to stimulate pistachio root growth and to control *Phytophthora drechsleri* through the production of auxin and other metabolites [56]. Beneficial effects of *Pseudomonas* spp. against *Gaeumannomyces graminis* var. tririci in wheat [57], *Fusarium oxysporum* on tomato [58], and *Pythium ultimum* in many crops have been confirmed [59].

Discovering and identifying biocontrol agents that can be utilized and commercialized for use in farms and orchards are important challenges in biocontrol research throughout the world. Plant probiotic bacteria of particular importance in biocontrol programs are those that produce a relatively wide range of metabolites against more than one disease pathogen on a particular host plant, while promoting growth and inducing resistance. Probiotics with these abilities can be considered for the production stages of biocontrol programs (including fermentation, formulation, and packaging). Similarly, these bacteria must also be capable of ready establishment in the rhizosphere or phyllosphere as a necessary condition for their use as biocontrol agents.

Finding appropriate isolates of potential biocontrol agents is very difficult and requires extensive and careful research. The biocontrol agent, in addition to being effective, must also be convertible into efficient formulations that can be mass-produced and widely used. The final product formulation is the culmination of knowledge of both fertilizer production technology and biocontrol agents. Therefore, the survival of microorganisms in the final product destined for sales shelves is of special importance [60]. Bacterial encapsulation is a practical technology that can protect the plant probiotic bacterial cells and guarantee their prolonged survival by their gradual release following addition to the soil. Encapsulated biocontrol bacteria are therefore more likely than free-living bacteria to be effective at controlling plant pathogens by virtue of their longer survival in unfavorable environmental conditions [61].

## 3. Biological Control of Pests

Since the time humans first began to produce food, insects have been one of the main obstacles to the production of more and better products. Humans have always tried to fight insects in many different ways, beginning with mechanical and agricultural management and later resorting to chemical methods. The subsequent recognition of the harmful effects of chemicals on both pests and beneficial insects has prompted the use of biological methods to control pests, with a focus on the use of microorganisms, such as fungi, bacteria, and viruses, that cause disease in specific host insects. This use of microbes against insects has led to the consideration of these organisms as microbial insecticides.

Biological control agents or biological pesticides are biological- and biochemical-based products that are employed to control plant pests and are considered next-generation plant protection products [63]. Biological pesticides, like chemical pesticides, are plant protection products used in the management of pests, plant diseases, and weeds, and they can be applied as necessary. Biological pesticides have various classifications, with most sources classifying them using the term “biological control agents,” which is the common term for all living controlling agents, including microorganisms [64]. Biological pest control has been conducted for more than 50 years in Iran; a prime example is the control of weevils using ladybugs in the northern regions of the country. Biological control is generally divided into two types:

1. Classical biological control: This method unleashes a natural enemy of a pest species in a new area; and
2. Applied biological control: This method uses native natural enemies and natural enemies stationed in an area affected by a particular pest species.

### 3.1. Advantages and Disadvantages of Using Biological Control Agents

Advantages: Biological control agents (1) have high potential for pest control; (2) have no or minimal effect on beneficial insects; (3) cause no resistance in insects; and (4) leave no contaminants in the environment. Disadvantages: Biological control agents (1) have gradual effects; (2) have a slow effect; (3) do not kill their host pathogens because that would destroy the biological control agents as well; (4) are often unpredictable because they are affected by many factors; (5) are difficult and costly to create and use; and (6) need expert supervision [65].

The first record of controlling pests using natural enemies dates back to 900 AD, when *Oecophylla smaragdina* F. was used to control insects that fed on the foliage of orange and tangerine trees [64,65,66]. In the 19th century, an Australian insect, *Icerya purchase,* abounded in California. American entomologists used a ladybug beetle, *Rodolia cardinalis,* to control the Australian insect’s population growth and promote its decline in the region [67].

Biological pesticides or biological control agents can be divided into four main groups [68]:

1. Microorganisms (microbial pesticides), including fungi, nematodes, viruses, bacteria, and protozoa. These contain more than 100 products that play important roles in integrated pest management, organic agriculture, and even traditional agriculture. Insects, weeds, pathogens, and nematodes, like other living organisms, suffer from diseases caused by microorganisms. In some cases, these pathogens are isolated, cultured, and mass-produced to control pests. These products are also called microbial pest control agents;
2. Products of natural origin, such as products derived from plants or microorganisms. More than 50 products of natural origin have been registered currently. Plant-derived pesticides are also called botanical pesticides. These pesticides are alkaloids or glycosides found in the flowers, stems, roots, or even seeds of plants;
3. Microbial pest control agents. These occur naturally or are genetically modified agents that are distinct from conventional chemical pesticides due to their unique mode of action, low consumption volume, and specificity for target species. These agents are used to control plant pathogens, pests, and weeds; and
4. Bacterial insecticides: These are one type of biological insecticides that eliminate plant pests by causing disease and death at different stages of insect growth [69]. One of the well-known bacteria that infects and destroys many harmful insects in the larval stage is *Bacillus thuringiensis*, abbreviated Bt. This bacterium is identified by a central, elliptical spore, as well as by a crystal. It was discovered in 1901 when the Japanese scientist Ishibata separated a parallel spore bacterium from a sick silkworm; he named it *Soto bacillus* [70]. In 1911, Berliner also separated the bacterial mashbebi from *Ephestia elutellain* in the Thuringia region of Germany and named it *Bacillus thuringiensis*. This bacterium is completely unique and does not cause disease in beneficial insects, humans, or other vertebrates [71].

### 3.2. Mode of Action of Bacillus thuringiensis

Larvae become infected with this bacterium via the gastrointestinal tract. The digestive systems of the larvae become paralyzed, and eventually the larvae cease to feed on the agricultural products. Symptoms: Insects killed by Bt toxin rapidly become discolored, darkened, and very soft. The internal tissues and organs are rapidly destroyed and become viscous, sometimes accompanied by a foul odor. Shortly after death, large amounts of bacteria are produced inside the insect’s body. The carcasses of the wrinkled insect larvae become dry and hard [72].

Bt biological insecticide has been formulated and manufactured in Iran [73]. Not only does it have none of the unwanted side effects of chemical insecticides, it is also not harmful to humans and the environment, and it has a high durability in the environment. This product can be used in biological control and integrated pest management (IPM) programs against the larvae of several plant pest moths.

### 3.3. The Technical Specifications of Bt Formulations

Active ingredient: Spiro crystal mixture—Effective substance: 4.8%, number of inert materials including preservative, wetting, adhesive, suspending, and diluting: 95.2%. Number of spores: about 100,000,000 per gram of dry matter. Biologically effective unit: (IU) 15,000 international units per mg. Ultraviolet (UV) protective coating of spores: starch microcapsules, ALG, gum arabic, etc. Physical state: Water-soluble powder, creamy white in color, with a size of 120 µm. Scope of pesticide: Early larvae of several butterfly pests: *Helicoverpa armigera, Tuta absoluta, Plutella xylostella, Culex quinquefasciatus*, and types of leaf-eating larvae. Mechanism of pesticide: poisoning of the larvae through digestion. After the larvae feed on plant organs sprayed with Bt, its crystals and spores are activated in the digestive tract of the larvae. After 5–7 days, the bacteria cause death of the insect due to starvation and bleeding in the gastrointestinal tract. Yield: The amount of Bt recommended for controlling early age larvae is 2–3 kg/ha. The effects of this insecticide are reduced in older larvae unless the dose is increased. Side effects: Bt has no adverse effects on humans, the environment, aquatic animals, or invertebrates. It does not harm non-target insects, such as parasites and predators, due to its specialized nature. Other research shows that this insecticide rarely causes resistance in the target pest [73]. Durability in the environment: Because of the protective coating, the maximum durability in nature is 5–7 days. Therefore, in populations with high levels of target pests, the recommendation is to repeat foliar application after 7–10 days, if there is generational interference. Shelf life: Depending on the type of formulation (water-suspended powder), it can be stored for two years in dry and cool conditions (4–10 °C) without loss of quality. However, the number of active spores decreases after one year under normal conditions, to the extent that the amount of active ingredient decreases to about half of the original amount after two years. Interference with chemical insecticides: can be used in combination with most chemical insecticides with a pH lower than 7 in the integrated management of IPM pests [74].

Other bacterial microbial toxins in addition to Bt have a very good and effective performance, but various factors negatively affect their efficacy, including UV in atmospheric conditions, washing off from the plant surface, and lack of vegetation. The negative effects of these factors can be minimized by encapsulation of Bt and other microbes to control pests.

Various polymers, such as gelatin, ALG, chitosan, starch, gum arabic, gellan gum, and milk protein, are used for the production of encapsulated biocontrol bacteria. Among these, ALG is a commonly used and practical polymer for the encapsulation of plant probiotic agents [75].

## 4. Bioencapsulation

PGPR strains should be viable, maintain a proper level during their shelf life until use, and retain strong viability in the soil [76]. The formulation of an efficient microbial can determine how successfully a biological agent will function [77]. Microencapsulation is a technology for packaging substances into small capsules that are capable of releasing these substances at controlled rates under certain conditions [78]. Bioencapsulation is an effective formulation that protects the microorganisms in the soil and controls their sustained release [11]. 

These formulations can be improved in two ways: by supplying nourishment for microbial growth and by using bio-composite capsules that can increase the numbers of encapsulated bacteria inoculated. Bioencapsulation of microbial inoculants has many advantages, such as protecting them in the soil against mechanical stresses and adverse environmental conditions, providing controlled release of the microorganisms, and decreasing pollution during transportation and storage [79]. Biopolymers (polymers produced by biological organisms) have been used for encapsulation in different industries and especially in agriculture. One example is *Pseudomonas putida* Rs198 encapsulated in ALG-bentonite; the encapsulated microbe has a better survival rate and effective colonization [80]. Gagne-Bourque et al. [81] have successfully encapsulated *Bacillus subtilis* B26 in an ALG-pea protein capsule.

Due to the increasing demand for microbial biocontrol agents, studies on new formulation techniques, and especially on the production of biological capsules, have increased significantly in recent years [82]. However, the interactions of the capsule matrix with the physiochemistry of soils are not well understood, and little information is available regarding the interactions with the agricultural ecosystem. Nevertheless, chitosan has been reported recently to have fungicidal activity [83].

A new range of second-generation Bt-based biotoxins with many benefits has been proposed as a unique result of genetic modification [84]. *Pseudomonas fluorescens* and Bt have been bioencapsulated for use against pests. Comparison of mycophenolic acid bioencapsulation and Bt toxin revealed that coated bioencapsulated cells contained one toxin while Bt has several toxic triglycerides. The advantage of bioencapsulation is that the most effective single toxin can be selected and expressed at high levels. A biological capsule consisting of single-gene Delta products (the endotoxins isolated from Bt) within killed *Pseudomonas* cells provided a more durable system for use against *Plutella xylostella* [85]. Bioencapsulation and the use of optimal pesticides leads to greater compatibility compared to conventional Bt insecticides when used against *Earias insulana*.

Another bioencapsulation protocol consists of dripping a mixture of rhizobacterium cells enclosed in ALG grains and starch into calcium [86]. The use of trehalose in the culture medium in addition to starch, as well as altering the growth stage of the cells, improved survival of the rhizobacteria during the process of bioencapsulation. This emphasizes the importance of selecting suitable methods to enhance the encapsulated agents’ viability while they are being produced and afterward during storage [86]. Some critical views on bioencapsulation have been pointed out by different authors [86,87].

Clearly, improving cell survival during bioencapsulation is not an easy task, and the effects of various factors, including the combination of growth, environment, and pressure on cells, will depend on the cells’ physiological condition and the parameters used for the bioencapsulation.

## 5. Methods for Encapsulation of PGPR

Various methods are applied to encapsulate bacterial cells, with extrusion, spray drying, and emulsion being the most important [88].

### 5.1. Extrusion Technique

**Figure 3:**
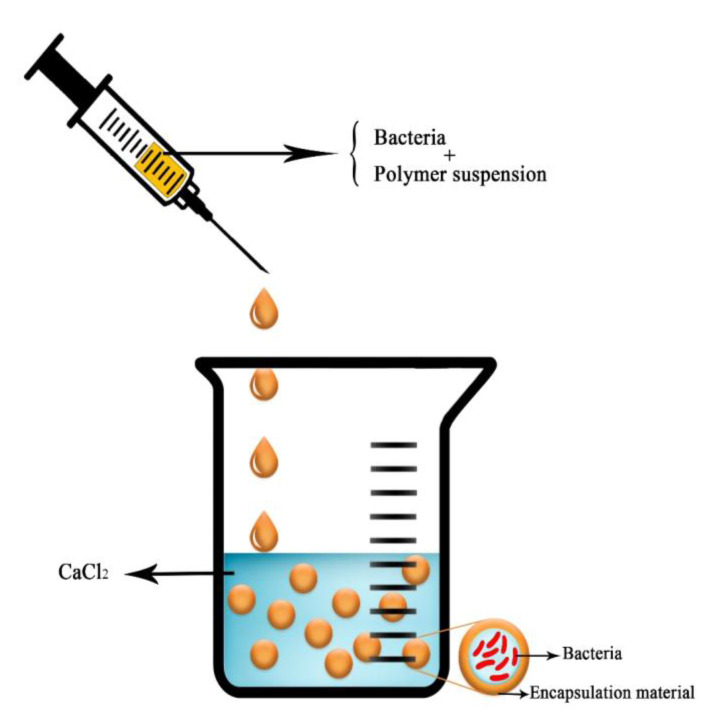
Schematic picture of the extrusion technique for bacteria encapsulation.

Encapsulation by an extrusion technique involves alteration of the wall material and the active material by intense pressure (Figure 3). This is one of the simplest and most efficient techniques for microbial encapsulation, for different reasons. Encapsulation in hydrogel-based biopolymers increases the efficiency of PGPR [89]. The interior pore of the extrusion device pumps the core material (the bacterial suspension), and the exterior pore pumps the wall substance to create a co-extruded rod made of core material and limited by wall substance. Drops from the created rod are molded into capsules while the system is spinning [90]. A benefit of the extrusion technique is that it imparts stability against oxidation [91].

Extrusion is an economical commercial method that is commonly used for the encapsulation of microbial agents, and it produces high-quality products. Saberi-Riseh and Moradi-Pour [28] used the extrusion method for encapsulation of *Bacillus subtilis* Vru1 using ALG-bentonite and found that this formulation was capable of controlling rot disease in bean root ascribed to *Rhizoctonia solani*.

### 5.2. Spray-Drying Technique

**Figure 4:**
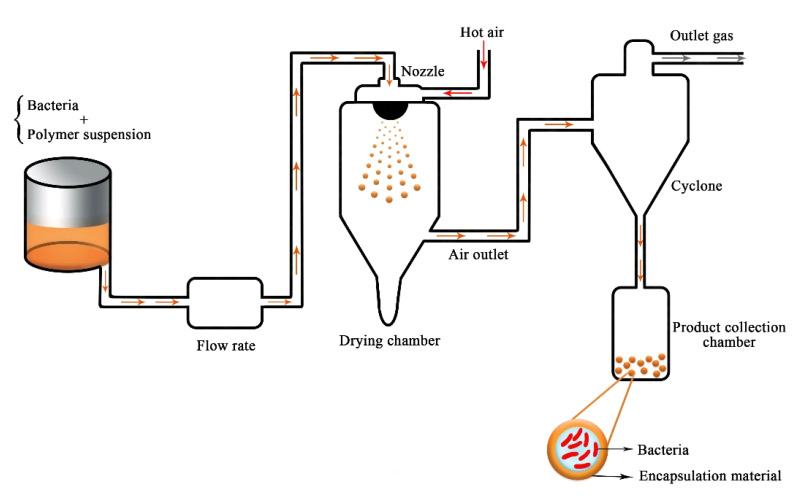
Schematic depiction of the spray-drying technique for bacteria encapsulation (adapted from [96]).

This technique involves the dispersal of microbial cells in a wall substance that forms an emulsion. Homogenized material is atomized and sprayed into a hot chamber; this process causes the solvent to vaporize, leaving microcapsules [92]. Spray-drying is a common commercial method and is used for substantial microbial formulations (Figure 4). It is an economical technique and produces a high-quality product [93]. For this method, the wall materials include slightly viscous substances that have effective drying attributes and high water solubility and are supplied at high concentration [94]. The advantages of this method are its low cost, the excellent quality of the produced capsules, and their quick solubility, high stability, and small size. However, since this process occurs in a hot environment, it might not be suitable for bacterial encapsulation. Other disadvantages of this method are the restriction in the selection of wall materials and the lack of microcapsule uniformity [95]. This method also produces a very fine powder, and it is unsuitable for heat-sensitive material. Saberi-Riseh and Moradi-Pour [95] reported that the number of *Streptomyces fulvissimus* Uts22 bacteria in chitosan-gellan gum microcapsules obtained by this method was approximately 10^8^ CFU g^−1^ after storage for 2 months.

### 5.3. Emulsion Method

**Figure 5:**
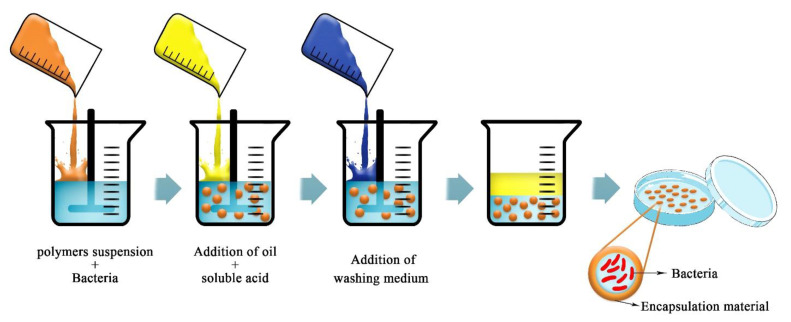
Schematic picture of the emulsification technique for bacterial encapsulation (adapted from [99]).

Various industries, such as pharmaceuticals, food, and agriculture, typically use material with high water solubility for their applications, and many of them use a water-in-oil emulsion to produce microcapsules [97]. With the emulsion method, small amounts of biopolymer suspension, such as ALG, gums, or gelatin, and amounts of pure oil are combined (Figure 5). Microcapsules differ in size according to the type of emulsification and the agitation speed. Moradi-Pour et al. [26] used ALG to make emulsions with soybean oil for encapsulation of *Pseudomonas fluorescens* VUPF5. ALG was selected for its high solubility in water in comparison to other biopolymers and for its capability for emulsifying a suspension of oil in water [98].

## 6. The Effect of Alginate Microcapsules of Bacillus Thuringiensis on Pests

Biocontrol agents have been proposed to resolve the environmental hazards posed by chemical pesticides. One of these promising solutions is the use of insecticidal bacteria, such as Bt. *Bacillus thuringiensis* secretes protein crystals during the sporulation phase that are toxic to insects like *Lepidoptera, Hymenoptera, Coleoptera,* and *Diptera* [100]. However, Bt has a poor performance on its own, and its durability in the environment and against UV radiation is low [101]. Therefore, scientists are trying to encapsulate Bt to increase its effectiveness. The mortality of *Tuta absoluta* (Meyrick) larvae on *Lycopersicon hirsutum* f. glabratum was achieved by Bt. Similarly, application of Bt to tomato leaves increased plant resistance and induced mortality at all ages of *T. absoluta* [102].

First instar larvae scratch the leaf for about 20 ± 45 min (before entering the mesophyll) so they have more access to Bt sprayed on the leaf, whereas second instar larvae have less access [103]. Third instar larvae are exposed to higher doses because they feed on more leaves, and the mortality rate among them is high. In fourth instar larvae, the reduction in mortality compared to older larvae is probably due to less toxin use.

To control the larvae of *Spodoptera exigua* (Hübner), celery leaves were impregnated with Bt, and the larvae were killed [104]. Bt was also used against the larvae of *Choristoneura rosaceana* (Harris) [105]. Research demonstrates that Bt does not have a strong performance by itself and that encapsulation can bring about better efficacy. ALG and gelatin were selected as Bt insecticide carriers due to their non-toxicity. The effect of ALG with Bt on biology and mortality was investigated in vitro in *Martianus dermestoides* [30]. Encapsulation of Bt reduced the UV degradation and increased the high-temperature resistance of the Bt toxin. The toxicity of ALG-encapsulated Bt was higher than without capsules. The extrusion method was used to prepare and describe ALG-encapsulated Bt microspheres. ALG can protect the bacterium from UV rays, so that its bacterial properties are not destroyed by UV radiation. The encapsulation of Bt powders in calcium-ALG capsules was also successfully performed and was found to increase the stability and durability of Bt for use in the agricultural industry. The percentage of control obtained for *Martianus dermestoides* was 95% [30].

Another study investigated Bt encapsulation efficiency against *Aedes aegypti* larvae using starch, maltodextrin, and corn-flour by spray encapsulation, and showed that Bt prevented the growth of microorganisms for a prolonged period [106]. This modulation may increase nutrition and consequently increase efficiency against *Ae. aegypti* larvae. This formulation was used against third instar larvae of *Ostrinia nubilalis* (*Hübner*), and led to a 90% mortality in the larvae [107]. Encapsulation of Bt with starch preserved the biological effects on *O. nubilalis* (Hübner), as the larvae swallowed and excreted the granules [108].

Many factors affect system performance, including humidity, concentration, dosage, insect age, and duration of exposure. The difference in the body size of the larvae indicates that even consuming a small amount of Bt reduces nutrition [109]. However, an acceptable dose for population reduction has not been obtained. One problem that needs to be taken into account is that if this starch formulation is left moist for a long time, it will be attacked by microorganisms and become moldy. To reduce this possibility, 1% calcium propionate was added to the formulation, and the aforementioned pest still showed a positive reaction to it [109]. Another formulation for encapsulating Bt contained several other substances, such as sunflower oil, ethanol, water, iron oxide nanoparticles, and acrylic particles. Laboratory encapsulation was tested on *Trichoplusiani* larvae [110]. The larval population diminished remarkably after 12 days, and the efficiency of the microencapsulated formulation was similar to that of a chemical pesticide [111].

Bt encapsulation in micro lipid droplets was utilized to control larvae of *Anopheles freeborni* and *Aedes aegypti* mosquitoes [112]. Bt crystals are heavier than water molecules and do not settle on the surface of the water. Therefore, *A. freeborni* larvae are less exposed to Bt crystals, as these mosquitoes live on the surface of the water.

Bt encapsulation in micro lipid drops is a system that can be used to store the desired bacteria and therefore will have a long-term effect [113]. Recent advances in liposome technology reveal that liposomes with various auxiliary additives can be designed to encapsulate Bt for testing against different mosquito larvae [114]. In addition, antimicrobial agents can be included to protect against the attack of other microorganisms on the rich set of fats and proteins.

We conclude that, regardless of the applied method, the encapsulation of the Bt bacterium as a treatment for various pests maintains its stability against environmental factors and increases its performance.

## 7. Application of Encapsulation Technology to Control Plant Disease

**Figure 6:**
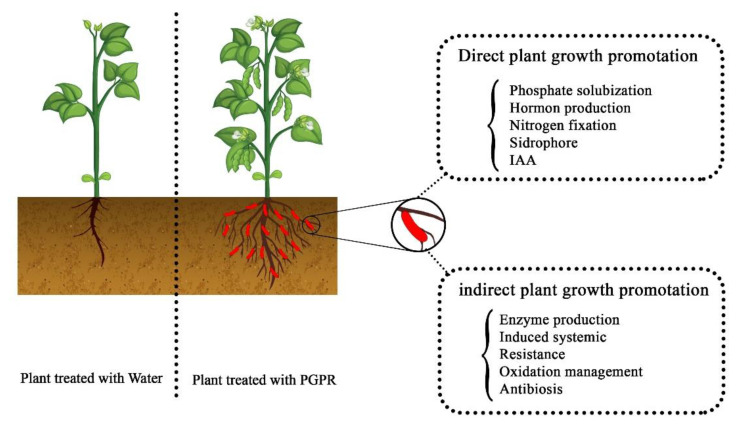
Interaction between plants and plant growth-promoting rhizobacteria.

Huge numbers of plant pathogens infect crops and reduce crop yields, with effects on agricultural products that can range from mild symptoms to disaster [115]. Controlling plant diseases is difficult because the pathogens have various populations in time, space, and genotype [116]. Lately, an explosion has occurred in the use of beneficial microorganisms that help plants grow better and control their diseases [117]. Plant growth can be stimulated by PGPR through siderophore production [118], nitrogen fixation [119], synthesis of auxin [120] and cytokinin [121], promotion of Acc deaminase [122], and production of vitamins and other plant hormones (Figure 6). Bacteria are sensitive to environmental conditions, such as temperature fluctuations, pH, humidity, competition, etc. [123]; therefore, protecting them from these factors inside a biodegradable covering is a promising plan. Encapsulation with biopolymers [124] can protect bacterial cells against environmental conditions and toxic compounds [87] and improve their PGPR activities, thereby leading to maximum cell viability and a subsequent increase in bacterial colonization around the plant roots.

ALG gel is non-toxic, inexpensive, biodegradable, and environmentally compatible, so it has been employed as the main material for encapsulation and bacterial immobilization [125]. The coating of bacteria within biodegradable capsules aids in the retention of bacterial cells within the soil [126]. Over the past few years, PGPR have received increasing consideration among many agricultural researchers owing to their great effectiveness in growth stimulation; they have also been successful in making plants resistant to pathogens [127,128]. Many researchers have claimed that encapsulation of ALG-based bacterial agents has great promise for biocontrol of plant pathogens. Moradi-Pour et al. [61] showed that the nanoencapsulation of *Bacillus subtilis* and *Pseudomonas fluorescens* together with carbon nanotubes and silica nanoparticles increased the proliferation and root length in micropropagated UCB1 pistachio plants. Kim et al. [11] investigated ALG encapsulation and the biocontrol agent *Pantoea agglomerans* strain E325 for gradual release against *Erwinia amylovora* in apple. This research confirmed the successful application of an encapsulated biocontrol bacterial agent, *P. agglomerans* E325, against *E. amylovora*, and it has been used as an effective strategy for plant disease management.

*Klebsiella oxytoca* Rs-5 was encapsulated in ALG, and its efficiency under salinity stress was evaluated. The findings reveal that encapsulated cells were significantly involved in the growth promotion of cotton plants compared to free bacteria under salty conditions [129]. Moradi-Pour et al. [26] reported that ALG/gelatin microcapsules of *Pseudomonas fluorescens* (VUPF5 and T17-4 strains) increased the growth rate of potato plants and significantly reduced potato dry rot disease. According to the observations of Saberi-Riseh and Moradi-Pour [28], plant growth was stimulated and seedling death was decreased in bean plants treated with ALG microcapsules containing *Bacillus subtilis* Vru1 enriched with titanium oxide (TiO_2_) nanoparticles compared to plants treated with uncoated bacteria. Tu et al. [29] indicated that the ALG/gelatin microcapsules of *Bacillus subtilis* SL-13 can serve as a novel microbial fungicide.

## 8. The Purpose of Co-Encapsulation Is to Manage Plant Pests and Diseases

Annual infestations of pests and plant pathogens cause significant economic damage to agricultural products. Unfortunately, the farmers’ first solution to this problem is to use chemical toxins, which can have many adverse effects on the environment. For this reason, researchers have proposed biological control agents as an effective alternative to chemical controls. Despite the development of high-capacity biocontrol agents around the world, the use of these agents in farms and orchards has, unfortunately, not had the desired results, mostly due to a lack of proper formulations that can maintain the survival of the biocontrol agents during storage and use. As shown by the information presented in this review and the demand for controlling plant pests and maladies, achieving new formulations, such as encapsulated biocontrol agents, can represent an important step toward minimizing the negative effect of environmental factors, thereby improving products for farm use, both qualitatively and quantitatively.

**Figure 7:**
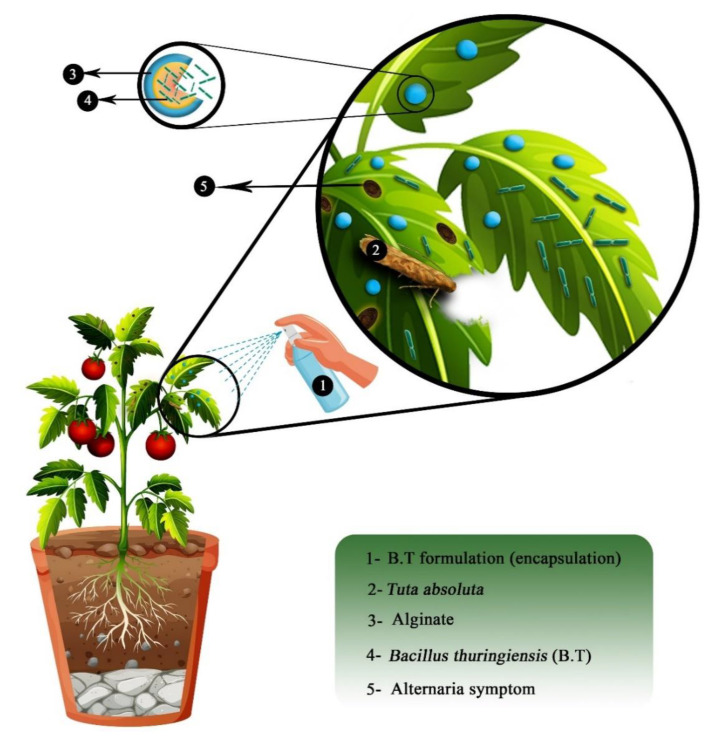
The effect of biocontrol bacteria microcapsules on biocontrol of pests and diseases.

The encapsulation of plant probiotic agents is a new technology in the field of agriculture, especially in the management of plant diseases, and is highly promising, particularly if important, economical, and available compounds such as ALG are used. This review has emphasized two important strategies:

1. Co-encapsulation of Bt bacteria (to control pests) and plant growth-promoting bacteria, such as *Bacillus* and *Pseudomonas* (to deal with plant maladies), in a biodegradable polymer coating, especially ALG (Figure 7); and
2. Finding a suitable biocontrol bacterium that can simultaneously control plant pests and diseases in formulations that can be used in agriculture.

## 9. Conclusions

The chemical pesticides frequently used to control plant pests and diseases have many destructive environmental effects. Today, several biotoxin products are commercially available and represent more suitable and sustainable alternatives for pest control. However, pest management practices using biotoxins have their own problems, including low efficacy in field conditions compared to chemical pesticides [130]. Research has therefore intensified in recent years regarding the production of new formulations and technologies that can increase the efficiency of biotoxins, especially formulations that encase Bt bacteria within efficient nanoparticles. This review demonstrates the attention this field has attracted in recent years and the progress made. The main goals of most research conducted on Bt-containing micro/nanoformulations have been increased effectiveness and continuity, longer durability of the formulations, and a resulting enhancement of plant growth, yield, and quality of agricultural products [131]. Although different techniques are used in the preparation of the current formulations, future work may require techniques that can create a multi-layered structure and enclose the biological control agent within a rigid and impenetrable coating.

Researchers have used a variety of polymers and different methods for the encapsulation of beneficial bacteria. They have also provided innovative ideas for increasing eco-friendly techniques for the efficient delivery of biocontrol bacteria. The technology of encapsulation with ALG can have a great effect in agriculture by immobilizing biologically relevant bacteria and active substances. The advantages of using ALG in encapsulation technology include the production of biocontrol bacteria microcapsules that can be as efficient as bio-fungicides, bio-pesticides, and/or bio-fertilizers in the agricultural fields. Since plant pests and diseases cause economic damage to many agricultural products every year, the use of the formulation proposed in this review, which is a combination of two bacteria for integrated pest and disease management, can be economically important for farmers. Using this type of formulation and controlled release of biological agents, in addition to stimulating essential elements that help plants thrive, can achieve pest and disease control in the host plant and increase the quality and quantity of agricultural products. The possibility of managing plant pests and diseases with beneficial bacterial agents emphasizes the necessity of deriving encapsulation formulations that can provide both proper shelf life and effective release of these agents.

## Figures and Tables

**Table 1:** Microbial immobilization in alginate capsules mixed with other coating materials.

Coating Material	Immobilized Cell	References
ALG–Poly-L-lysine	*Lactobacillus acidophilus* 547	[19]
ALG–Chitosan	*Penicillium purpurogenum*	[20]
ALG–Chitosan	*Acetobacter sp.* CCTCC M209061	[21]
ALG–Chitosan	*Saccharomyces cerevisiae*	[22]
ALG–Chitosan	*Lactobacillus bulgaricus*	[23]
ALG–Protamine	*Lactobacillus casei* CICC 23185	[24]
ALG–Whey protein	*Pseudomonas fluorescens* VUPF506	[25]
ALG–Gelatin	*Pseudomonas fluorescens* VUPF5	[26]
ALG	*Pantoea agglomerans*	[27]
ALG–Bentonite-Starch	*Bacillus subtilis* VRU1	[28]
ALG–Gelatin	*Bacillus subtilis* SL-13	[29]

**Table 2:** *Bacillus thuringiensis* microcapsules produced with alginate in combination with other polymers.

Pest	Coating Material	Reference
*Martianus dermestoides*	ALG	[30]
*Ostrinia furnacalis*	ALG–Starch–Gelatin	[31]
*Ephestia kuehniella*	ALG–Starch–Gelatin	[32]
*Culex quinquefasciatus*	ALG–Hollow glass beads	[33]
*Heliothis virescens*	ALG	[34]
*Spodoptera littoralis*	ALG	[35]
*Spodoptera frugiperda*	ALG–Corn oil	[36]
*Plutella xylostella*	ALG–Gelatin	[37]

## Data Availability

Not applicable.
